# Combined Lifestyle and Herbal Medicine in Overweight Women with Polycystic Ovary Syndrome (PCOS): A Randomized Controlled Trial

**DOI:** 10.1002/ptr.5858

**Published:** 2017-07-07

**Authors:** Susan Arentz, Caroline A. Smith, Jason Abbott, Paul Fahey, Birinder S. Cheema, Alan Bensoussan

**Affiliations:** ^1^ National Institute of Complementary Medicine Western Sydney University Building 5, Campbelltown Campus, Locked Bag 1797 Penrith NSW 2751 Australia; ^2^ School of Women's and Children's Health, Level 1, Women's Health Institute, Royal Hospital for Women University of New South Wales Barker Street Randwick NSW 2031 Australia; ^3^ School of Science and Health Western Sydney University Building 24, Locked Bag 1797 Penrith NSW 2751 Australia

**Keywords:** polycystic ovary syndrome, overweight, herbal therapeutics, lifestyle, menstrual regulation, safety

## Abstract

Polycystic ovary syndrome (PCOS) is a common, complex reproductive endocrinopathy characterized by menstrual irregularities, hyperandrogenism and polycystic ovaries. Lifestyle modification is a first‐line intervention; however, there are barriers to success for this form of self‐care, and women often seek adjunct therapies including herbal medicines. This pragmatic, randomized controlled trial, delivered in communities of Australia in overweight women with PCOS, compared the effectiveness and safety of a lifestyle intervention plus herbal medicine against lifestyle alone. All participants were helped to construct a personalized lifestyle plan. The herbal intervention consisted of two tablets. Tablet 1 contained *Cinnamomum verum*, *Glycyrrhiza glabra*, *Hypericum perforatum* and *Paeonia lactiflora*. Tablet 2 contained *Tribulus terrestris*. The primary outcome was oligomenorrhoea/amenorrhoea. Secondary outcomes were hormones; anthropometry; quality of life; depression, anxiety and stress; pregnancy; birth outcomes; and safety. One hundred and twenty‐two women gave their consent. At 3 months, women in the combination group recorded a reduction in oligomenorrhoea of 32.9% (95% confidence interval 23.3–42.6, *p* < 0.01) compared with controls, estimated as a large effect (η_p_^2^ = 0.11). Other significant improvements were found for body mass index (*p* < 0.01); insulin (*p* = 0.02) and luteinizing hormone (*p* = 0.04); blood pressure (*p* = 0.01); quality of life (*p* < 0.01); depression, anxiety and stress (*p* < 0.01); and pregnancy rates (*p* = 0.01). This trial provides evidence of improved effectiveness and safety for lifestyle intervention when combined with herbal medicines in women with PCOS. © 2017 The Authors. Phytotherapy Research published by John Wiley & Sons Ltd

## Introduction

Polycystic ovary syndrome (PCOS) is a complex reproductive and endocrine disorder, characterized by heterogeneous presentation of menstrual irregularity (oligomenorrhoea/amenorrhoea), hyperandrogenism, polycystic ovaries, metabolic and psychological disorders and affects up to 18% of reproductive aged women (March *et al*., 2010). The PCOS represents a major health burden for women and communities (Teede *et al*., 2010) with economic costs in America conservatively estimated at $US4.36bn per year (Azziz *et al*., 2005).

A multidisciplinary approach is emphasized in the evidence‐based guidelines for management of PCOS with lifestyle intervention as first‐line treatment, which may regulate menstruation, reduce hyperandrogenism, treat hyperinsulinaemia and improve quality of life (Azziz *et al*., 2005; ESHRE, 2012). However the strength of evidence for lifestyle intervention is limited by high attrition in randomized controlled trials (RCTs) (Moran *et al*., 2013a), and clinical uptake remains impeded by the lack of evidence for optimal dietary and exercise practices (Azziz *et al*., 2005). In addition, physical and psychosocial barriers are commonly observed in overweight women, particularly those with established obesity (Moran *et al*., 2013b). Additional forms of management include pharmaceuticals (oral contraceptive pills and metformin) (ESHRE, 2012); however, these have limited capacity to address the range of PCOS symptoms (Diamanti‐Kandarakis *et al*., 2003) and are often contra‐indicated because of increased risk of co‐morbidities (Diamanti‐Kandarakis *et al*., 2003; ESHRE, 2012) or have high adverse effect profiles (Legro *et al*., 2007a, 2007b, Tang *et al*., 2012). Furthermore, few women are satisfied with pharmaceutical management. In a survey of women with PCOS, 99% (648 of 657) expressed their desire for effective treatment alternatives to birth control pills and fertility drugs, (Sills *et al*., 2001) and as many as 70% of women with PCOS use medical treatment adjuncts such as complementary medicines (Arentz *et al*., 2014a, 2014b).

Approximately two out of five women with PCOS report use of herbal medicine (Arentz *et al*., 2014a, 2014b). Plant‐based medicines contain biologically active chemicals that may alter reproductive endocrinology in women with PCOS (Arentz *et al*., 2014a, 2014b), and metformin, one of the primary pharmacological agents indicated for treatment of women with PCOS, was originally identified in the herb *Galega officinalis* (Bailey and Day, 2004)*.* However, despite women's use, the evidence for the effectiveness and adverse effects of herbal medicine is scarce (Zhang *et al*., 2010a, 2010b, Arentz *et al*., 2014a, 2014b, Lai *et al*., 2017). Whilst there is evidence from RCTs for individual herbal extracts in women with PCOS, (Chan *et al*., 2006, Wang *et al*., 2007, Grant, 2010, Kamel, 2013, Kort and Lobo, 2014, Shahin and Mohammed, 2014), many studies have high risks of bias, and the use of single plant extracts is inconsistent with typical complex herbal prescribing practices (Vickers and Zollman, 1999, Mills and Bone, 2000, Greenlee *et al*., 2007, Sarris and Wardle, 2010, Zhang *et al*., 2010a, 2010b, Lai *et al*., 2017).

In light of the frequent use of herbal medicine by women with PCOS and the absence of robust evidence, examination of herbal medicine as it is used in the community was considered an appropriate research strategy due to its capacity to provide relevant answers to questions of women and clinicians (Fønnebø *et al*., 2007). We conducted an RCT to determine the clinical effectiveness of combining a herbal medicine treatment (including five herbal extracts) with a lifestyle intervention, compared with lifestyle alone for a greater reduction in oligomenorrhoea in overweight women with PCOS (Qublan *et al*., 2007, Thomson *et al*., 2008). Secondary outcomes were reproductive and metabolic hormones, anthropometric markers, quality of life, psychological outcomes and safety according to increased blood pressure and adverse reactions.

## Materials and Methods

### Study population

This study included women aged 18–44 years with PCOS with a confirmed medical diagnoses according to the Rotterdam criteria (ESHRE, 2004) and a body mass index (BMI) greater than or equal to 24.5 kg/m^2^ (within the category of overweight for women with PCOS (ESHRE, 2012)). Women taking oestrogens and/or progestogens to regulate menstrual bleeding or antidepressants including selective serotonin reuptake inhibitors, selective noradrenergic reuptake inhibitors, tetracyclic antidepressants, noradrenergic and selective serotonin reuptake inhibitors, monoamine oxidase inhibitors and melatonergic antidepressants were excluded from the trial because of potential negative pharmacokinetic interactions with these pharmaceuticals and the herbal medicine (Izzo *et al*., 2016, Linde *et al*., 2008). Women were recruited in New South Wales, Queensland and Victoria, Australia, using advertising and referrals from health providers, gynaecologists and through the social networking site Facebook. Participants provided written consent prior to baseline data collection. Our study recruitment took place in community settings including participants' homes and workplaces, gymnasiums, cafes and parks.

### Study design

This trial was designed pragmatically and intended to inform the contextual decisions of clinicians treating overweight women with PCOS for which lifestyle intervention is recommended as first‐line intervention (AAPCOS, 2011; ESHRE, 2012). Ethical approval of the study was obtained from the Western Sydney University Human Research Ethics Committee (EC00314) on 20 December 2011. An amendment to the trial protocol approved on 9 August 2012 enabled recruitment from electronic social media (Facebook), delivery of the interventions and collection of data at locations convenient to participants. Women meeting the eligibility criteria were randomized in parallel groups to herbal medicine plus a lifestyle intervention or the lifestyle intervention alone. The randomized sequence was computer generated in permuted blocks of 50 by an external, independent organization (Sealed Envelope Limited, London, UK) (http://www.SealedEnvelope.com), communicated via short message service in real time at the conclusion of baseline data collection and concealed from the research team. Three levels of stratification were defined as BMI 24.5 to 29.9, BMI 30 to 33 or BMI over 33. The aim of stratification was to minimize selection bias and prevent of women with similar BMI kg/m^2^ characteristics being allocated to either the test or control groups. Participants and clinicians were aware of group assignment and therefore not blinded; however, the analysis was undertaken blind to group allocation.

Two subgroups were defined *a priori* on the Australia and New Zealand Clinical Trial Registry in January 2012 (reference: 201126 12000 122 8532). These were (i) women who participated in blood tests and (ii) women who wanted to conceive. The subgroup of women who participated in blood tests included women with oligomenorrhoea, selected in the order of recruitment and limited by the funding available for this doctoral study. The subgroup of women wanting to conceive included participants who self‐identified during baseline data collection.

### Interventions

The interventions were administered for 3 months.

#### Both groups: lifestyle intervention

The lifestyle intervention was guided by the evidence‐based guidelines for the management of PCOS (Teede *et al*., 2010; AAPCOS, 2011) defined as dietary and exercise behaviours that induce weight loss or prevent weight gain. The evidence‐based guidelines recommend a calorie‐controlled diet within a healthy food choice setting (AAPCOS, 2011) and exercise for at least 150 min per week including 90 min of aerobic activity (60–90% of maximum heart rate). Two trained lifestyle coaches (a nutritionist and an exercise physiologist) collaboratively introduced diet modification (identification of nutrient dense foods, calorie content and low glycaemic index carbohydrates (Marsh *et al*., 2010, Moran *et al*., 2013a, 2013b)) and a structured sequence of aerobic and progressive resistance exercises. Women had access to the lifestyle coaches throughout the trial, were contacted fortnightly, invited to attend supervised exercise sessions and helped to construct their own personalized lifestyle plan. As a pragmatic trial reflecting real‐world conditions, the lifestyle programme and its delivery were allowed to vary between participants with the aim to maximize generalizability of clinically relevant findings (Rothwell, 2005, Zwarenstein *et al*., 2008). In the event of pregnancy, participants were advised to continue the lifestyle intervention without increasing the intensity of exercise until consultation with their obstetric carer (McDonald *et al*., 2016).

#### Intervention – herbal medicine

Herbal medicine was administered in the form of two types of herbal medicine tablets each containing different herbal ingredients and two 30‐min consultations at trial weeks 4 and 8. During the consultations, women were asked about their well‐being and assessed for adverse effects by a qualified naturopath.

The new herbal regimen was trialled following clinical trial notification with the Therapeutic Goods Administration, Australia. The herbal ingredients were selected based on the evidence for reproductive endocrine effects (Arentz *et al*., 2014a, 2014b) and tailored to wider presentations of PCOS guided by naturopathic principles indicated by commonly observed clinical signs and symptoms of overweight women with PCOS (ESHRE, 2012).

Tablet one was manufactured specifically for the trial and contained the combined extracts of *Glycyrrhiza glabra*, *Paeonia lactiflora*, *Cinnamomum verum* and *Hypericum perforatum* (Table S1). These individual extracts are commonly combined in western herbal formulations for treatment of women with PCOS (Mills and Bone, 2000). The *G. glabra* and *P. lactiflora* were included to potentially reduce androgens (Yaginuma *et al*., 1982, Takahashi and Kitao, 1994, Armanini *et al*., 2004, Armanini *et al*., 2007); *C. verum* was included to improve insulin sensitivity (Wang *et al*., 2007) and menstrual regularity (Kort and Lobo, 2014); and *H. perforatum* was included to reduce depression (Gaster and Holroyd, 2000). Although there was no evidence for *H. perforatum* specifically in women with PCOS, evidence from systematic reviews has demonstrated effectiveness for mild to moderate depression (Gaster and Holroyd, 2000, Linde *et al*., 2008), which is prevalent in women with PCOS. Additional actions of the herbal medicines included potential digestive carminative properties (*G. glabra* and *C. verum*), improved hepatic metabolism (*H. perforatum* and *G. glabra*), antiinflammatory actions (*G. glabra*) and improved mood and cognitive effects (*H. perforatum* and *P. lactiflora*) (Bone and Morgan, 1996, Mills and Bone, 2000, Greenlee *et al*., 2007, Asl and Hosseinzadeh, 2008, Wardle *et al*., 2012).Three tablets once a day were administered throughout the trial. Participants were advised to cease taking the herbal medicine tablets if they conceived because of unestablished safety of use of these herbal medicines in pregnancy (Izzo *et al*., 2016).

Tablet two, MediHerb Tribulus Forte (Integria Healthcare (Australia) Pty Ltd), contained *Tribulus terrestris* extract equivalent to 13.5 g aerial parts (Table S2). The *T. terrestris* was provided separately because of its possible potentiating effect of follicle stimulating hormone (FSH) in women (Arentz *et al*., 2014a, 2014b). Administration of this tablet was limited to the follicular phase of the menstrual cycle. Dosage of three tablets per day for ten consecutive days commenced on menstrual cycle day 5 for oligomenorrhoeic women and within 1 week of trial commencement for women with amenorrhoea.

The two herbal tablets were manufactured by MediHerb (Integria Healthcare (Australia) Pty Ltd). The contents of the newly formulated tablet were verified independent to the manufacturer (Singh *et al*., 2013). Liquid chromatography coupled with photodiode array detection and electrospray ionization with tandem mass spectrometry was utilized to confirm four herbal extracts and nine biomarkers against standards specified by the European Commission Directorate for Agriculture guidelines (2002). Tribulus Forte is a listed medicine on the Australian Register of Therapeutic Goods (ARTG 185079) and available following consultation from herbal practitioners. The tablets were manufactured in accordance with good manufacturing practice. Commercialization of the novel herbal tablet remains at the discretion of the manufacturer.

### Study outcomes

The primary and secondary outcomes were selected on the basis of empirical evidence from women with PCOS about the most important outcomes from shared decision‐making and risks associated with PCOS (Teede *et al*., 2010). Study outcomes were assessed at 3 months after adjustment for baseline menstrual cycle variation at baseline. The primary end point was oligomenorrhoea defined as irregular menstruation or 35 to 179 days between menstrual periods (ESHRE, 2012) measured as the mean number of days in the menstrual cycle. Pre‐specified secondary study end points included serum concentration of reproductive hormones; glucose and insulin sensitivity; anthropometric characteristics; health‐related quality of life (HRQoL) and depression, anxiety and stress; pregnancy and birth outcomes; and safety of the herbal medicine assessed as increased blood pressure and adverse reactions.

### Data collection

The primary outcome was assessed by the number of days in the menstrual cycle and was self‐reported using printed menstrual charts or documented using websites providing digital menstrual cycle tracking services (FertilityFriend, 2014). Hormones [oestradiol, FSH, luteinizing hormone (LH), testosterone, sex hormone binding globulin (SHBG) and free androgen index (FAI)] were assessed by registered pathology companies compliant with statutory standards specified by the Australian Government (NPAAC, 2013). Blood was collected between 2 and 10 days of the menstrual cycle for non‐amenorrheic women or within 1 week of trial commencement for women without menstrual cycles. Insulin sensitivity was assessed by insulin and glucose serum concentrations following 8 h fasting and with the Quantitative Insulin Sensitivity Check Index (Katz *et al*., 2000). Anthropometry was assessed by body weight (kg), BMI kg/m^2^, waist and hip circumference (centimetres) and waist‐to‐hip ratio (W:H): HRQoL was assessed by the Polycystic Ovary Syndrome Questionnaire (PCOSQ) (Cronin *et al*., 1998), depression, anxiety and stress were assessed by the depression, anxiety and stress short form (DASS 21) (Lovibond and Lovibond, 1995) and pregnancy and live birth outcomes were assessed by serum measurements of beta human chorionic gonadotropin (BHCG) β HCG concentration (following β HCG detection in urine), ultrasound reports and post‐natal reports provided by participants. Adverse effects were assessed as blood pressure increments measured at weeks 4 and 8 and participants self‐reporting adverse experiences, reactions or events (Therapeutic Goods Administration, 2000) throughout the trial to the clinicians. Participant compliance was assessed by tablet count with the return of contents of herbal bottles at 3 months. Women's compliance with the exercise and dietary intervention was assessed fortnightly and during an interview at week 12. Women self‐reported the intensity of exercise (mild, moderate or vigorous) and number of minutes per week, dietary compliance was assessed through the average number of self‐reported servings of vegetables and fruit per day and number of high energy, nutrient sparse meals per week. There were no penalties including discontinuation in the trial for non‐compliance.

### Sample size and statistical analysis

We estimated that a sample size of 110 women completing the study would have 80% statistical power (significance level α = 0.05) to detect a clinical difference determined *a priori* in the mean percentage reduction in the number of days in the menstrual cycle of 55% for the group receiving the combined intervention, compared with a reduction of 30% in the control group. This estimation was based on studies reporting similar outcomes in women with PCOS for lifestyle interventions (Hoeger *et al*., 2004, Qublan *et al*., 2007, Thomson *et al*., 2008). Based on attrition rates from other studies, we estimated a 25% loss to attrition and calculated the necessary sample size at 148. As the trial progressed, it became apparent that attrition was approximately 10% and the minimum sample size was recalculated to 122 participants.

Data were coded and entered onto statistical software spss (IBM, 2013) by an independent data entry clerk and statistically analysed blind to group assignment. There were no interim analyses. Analyses were performed on an intention‐to‐treat basis, and data from participants who withdrew or were withdrawn were analysed according to their group of random assignment (Fisher *et al*., 1990). Missing end point data were imputed with the last observation carried forward method. In alignment with the study protocol and stated *a priori*, subgroup analyses were conducted for women who participated in blood tests (*n* = 64) and women wanting to conceive (*n* = 70). Baseline characteristics were summarized using counts and percentages for categorical variables and means and standard deviations if continuous. The randomized groups were observed for baseline differences of a magnitude of 10% or more.

Analysis of covariance was used to investigate differences between groups at 3 months after controlling for baseline levels. Assumptions confirmed prior to analysis of covariance included homogeneity of variance (Levene's test), linear relationships, homoscedasticity and homogeneity of the regression slopes. Positively skewed data with less than 30 observations were converted to their logarithmic value prior to statistical testing and back converted prior to reporting results. The effect size was estimated using partial Eta squared $\left(\eta_{p}^{2}\right)$ where a value of 0.01 was interpreted as a small effect size, 0.02 to 0.06 as medium and 0.07 to 0.14 as a large effect size (Cohen, 1973). Results were presented as the adjusted mean difference (MD) between groups at end point and associated 95% confidence intervals (CIs). Differences between groups for secondary binary outcomes (pregnancy, miscarriage and live birth rates) were analysed using chi‐squared tests and relative risks (RRs). The effect of loss of body weight and on the primary outcome after controlling for baseline menstrual cycle variation and study group was examined with partial eta squared $\left(\eta_{p}^{2}\right)$.

### Results

**Figure 1:**
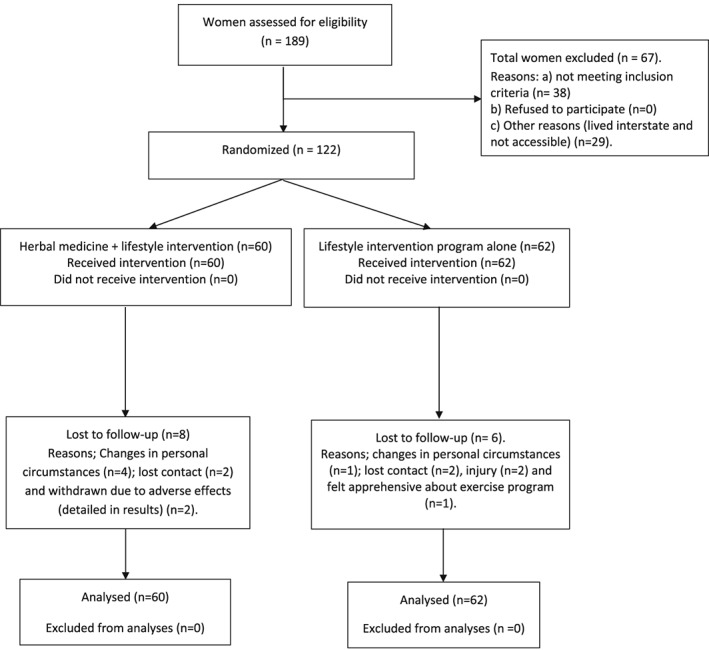
Flow chart of enrolment and analysis (CONSORT diagram).

One hundred and twenty‐two women met the inclusion criteria and were enrolled into the study between August 2012 and January 2014 (Fig. 1). Fourteen women (11.5%) withdrew or were lost from the trial at similar rates for each group. Two women in the treatment arm were withdrawn from the trial because of mild adverse events. These are documented in full in Fig. 1.

Randomization produced no imbalances between groups for demographic, reproductive, lifestyle characteristics or pharmaceutical use (Table 1). Participant characteristics were typical of overweight women with PCOS (ESHRE, 2012) with high prevalence of long and irregular menstrual cycles (77.9%), biochemical hyperandrogenism (48%), elevated LH, FSH ratio (35.8%), insulin resistance (51.7%), low quality of life (82%) and moderate‐to‐high depression, anxiety and stress. The mean (±standard deviation) number of days in the menstrual cycle for women assigned to the herbal medicine plus lifestyle was 106.0 (±123.0) days at baseline and for women allocated to the lifestyle intervention only group 109.5 (±148.0) days, (*p* = 0.889). Serum concentration of hormones was analysed for a subgroup of 64 participants. Seventy women were hoping to conceive during the trial.

**Table 1:** Baseline characteristics of participants by study group

Baseline characteristics	Herbal medicine plus lifestyle intervention (*n* = 60)	Lifestyle intervention alone (*n* = 62)	Difference between groups
Physical characteristics			
Mean age (years, ±SD)	29.2 ( 5.6)	28.9 (5.6)	0.4
Number aged 35 years or more (%)	11 (18.3)	8 (12.9)	5.4%
Mean weight (kg, ±SD)	93.2 (18.9)	97.3 (21.3)	4.1
Mean BMI (kg/m^2^, ±SD)	34.1 (7.2)	35.2 (6.8)	1.1
Mean waist circumference (cm, ±SD)	100.5 (13.3)	103.5 (17.1)	3.0
Mean waist‐to‐hip ratio (±SD)	0.83( 0.07)	0.85 (0.09)	0.02
Mean systolic blood pressure (mmHg, ±SD)	119.0 (11.7)	119.9 (11.9)	0.9
Mean diastolic blood pressure (mmHg, ±SD)	74.8 (13.1)	75.2 (14.3)	0.4
Ethnicity			
Caucasian (%)	40 (66.7)	43 (69.4)	2.7%
Asian (%)	4 (6.7)	2 (3.2)	3.5%
Aboriginal TSI (%)	3 (5.0)	0 (0.0)	5.0%
Polynesian (%)	2 (3.3)	2 (3.2)	0.1%
Maori (%)	1 (1.7)	0 (0.0)	1.7%
Unknown (%)	10 (16.7)	15(24.2)	7.5%
Education			
High school (%)	55 (91.7)	55 (88.7)	3.0%
Tertiary qualifications (%)	53 (49.1)	55 (50.9)	1.8%
Employment			
Full time (%)	36 (60.0)	32 (51.6)	8.4%
Part time (%)	10 (16.7)	11 (17.7)	1.0%
Student (%)	5 (8.3)	12 (19.4)	11.1%
Home duties (%)	6 (10.0)	5 (8.1)	1.9%
Casual employment part time (%)	3 (5.0)	2 (3.2)	1.8%
Reproductive characteristics			
Mean cycle length (days, ±SD)	106.0 (123.0)	109.5 (148.0)	3.5
Number with regular cycles (25–34 days) (%)	11 (18.3)	16 (25.8)	7.5
Number with oligomenorrhoea (35–179 days) (%)	40 (66.7)	36 (58.1)	8.6%
Number with amenorrhoea 180–1001 days (%)	9 (15.0)	10 (16.1)	1.1%
Number with normal serum testosterone (0.2–1.8 nmol/L) (%)^a^	15 (44.0)	19 (51.4)	7.4%
Number wanting to conceive (%)	34 (56.6)	36 (58.1)	1.5%
Mean time spent trying to conceive at trial entry (months ±SD)	22 (28.2)	18 (15.1)	4.1
Number with no prior conceptions (%)	42 (70.0)	41 (66.1)	3.9%
Number with null parity (%)	47 (78.3)	45 (72.6)	5.7%
Lifestyle characteristics			
Number of smokers (%)	7 (11.4)	5 (8.2)	3.2%
Number of vigorous exercisers (%)^b^	30 (50.0)	36 (58.1)	8.1%
Number exercising less than 150 minutes per week (%)	39 (65.0)	39 (62.9)	2.1%
Number consuming health conscious diets^c^	38 (63.3)	41 (66.1)	2.8%
Pharmaceutical use			
Number using ovulation induction (%)	1 (1.7)	2 ( 3.2)	0.8%
Number using insulin sensitisers (%)	11 (18.3)	11 (17.7)	0.6%
Number using herbal medicines (%)	34 (56.7)	28 (45.2)	11.5%

a Percentage calculated from the number of women in the subgroup of serum hormone measurement (*n* = 71).

b Vigorous exercise was defined as 60–90% of maximum heart rate and unable to engage in conversation.

c Health conscious diets defined as two servings of fruit and five of vegetables per day, energy sparse and low GI carbohydrates.

At follow‐up, participants reported improved lifestyle practices with 89 of 122 (73%) self‐reporting at least 150 min exercise per week and 105 of 122 (86%) reporting health conscious dietary decisions. There were no significant differences between groups for number exercising 150 min or more per week (43 of 60 in the lifestyle plus herbal group, compared with 46 of 62 in the lifestyle only group, RR 0.99, 95% CI 0.80 to 1.24, *p* = 0.98) and number reporting health conscious dietary choices (57 of 60 in the lifestyle plus herbal group compared with 54 of 62 in the lifestyle only group, RR 1.09, 95% CI 0.98 to 1.22, *p* = 0.12).

At 3 months, there was a statistically significant difference in oligomenorrhoea for women in the herbal medicine plus lifestyle group compared with controls adjusted for baseline cycle length (Table 2). The mean menstrual cycle length was 43 days (95% CI 21 to 65, *p* < 0.001) lower for women in the herbal medicine group than for those in the lifestyle only group. The treatment effect size was estimated as large $\left(\eta_{p}^{2}=0.11\right)$. The proportion of women with normal menstrual cycle length (20–34 days) in the herbal medicine plus lifestyle group was significantly greater compared with controls 33 of 60 (55.0%) compared with 15 of 62 (24.2%) (RR 1.9, 95% CI 1.32 to 2.69, *p* < 0.01).

**Table 2:** Menstrual cycle and clinical characteristics of overweight women with PCOS after administration of lifestyle intervention plus herbal medicine compared with lifestyle alone at three months

	Herbal medicine plus lifestyle *N* = 60	Lifestyle intervention programme *N* = 62	Difference between groups^d^				
	Mean	Mean	Adjusted + mean difference (SE)	95% CI		*P* value	Partial eta squared
Oligomenorrhoea							
Number of days between menstrual periods	63.7	106.6	−42.9 (11.1)	−64.8	−21.1	**<0.01**	0.11
Anthropometric characteristics							
Body weight in kilograms	90.2	97.2	−2.95 (0.8)	−4.4	−1.5	**<0.01**	0.12
Body mass index (BMI)^a^	33.0	35.0	−1.0 (0.3)	−1.6	−0.5	**<0.01**	0.11
Waist circumference (cm)	96.1	102.3	−3.41 (0.8)	−4.9	−1.8	**<0.01**	0.04
Waist to hip (W:H)^b^	0.82	0.84	0.001 (0.01)	−0.01	0.01	0.91	0.00
Reproductive hormones (menstrual cycle days 2–10)	*n* = 34	*n* = 37					
Follicle stimulating hormone (FSH) IU/L^c^	5.3	4.9	0.25 (0.4)	−0.5	1.5	0.53	0.01
Luteinizing hormone (LH) IU/L^c^	5.84	7.4	−1.82 (0.9)	−3.5	−0.1	**0.04**	0.06
FSH:LH ratio	1.03	1.02	0.10 (0.1)	−0.13	0.31	0.40	0.01
Oestradiol pmol/L^c^	217.0	148.1	68.9 (31.6)	5.5	132.3	**0.03**	0.08
Testosterone nmol/L	1.63	1.59	−0.04 (0.2)	−0.33	0.25	0.79	<0.01
Sex hormone binding globulin nmol/L	48.6	44.0	7.4 (7.2)	−7.0	21.8	0.31	0.02
Free androgen index %	5.4	4.9	0.23 (1.1)	−1.97	2.44	0.83	<0.01
Metabolic hormone concentration	*n* = 26	*n* = 25					
Fasting glucose nmol/L^c^	5.0	5.24	−0.44 (0.3)	−0.97	1.01	0.13	0.06
Fasting insulin mU/L^c^	12.3	20.3	−5.93 (2.5)	−10.9	−0.97	**0.02**	0.11
QUICKI^c^	0.32	0.35	0.002 (0.02)	−0.06	0.12	0.24	0.03
Adverse effects							
Systolic blood pressure	114.3	118.0	−3.6 (1.4)	−6.3	−0.9	**0.01**	0.06
Diastolic blood pressure	69.3	74.6	−5.13 (1.4)	−7.8	−2.4	**<0.01**	0.11

Quantitative Insulin Sensitivity Check Index (QUICKI) method = 1/(log(I_o_) + log G_o_): I_o_ fasting insulin levels in mIU/mL; G_o_ fasting glucose levels in mg/dL (conversion from mmol/L to mg/dL: multiplied by 18.0).

a BMI = bodyweight (kg) divided by height (metres) squared. (BMI = w(kg)/height(m)^2^).

b Waist‐to‐hip ratio = hip in (cm) divided by waist (cm). (Hip (cm)/waist (cm)).

c Log transformations were carried out on the data before analyses.

d All results are reported after adjustment for baseline values using ANCOVA.

p values <0.05

Secondary anthropometric outcomes at 3 months found a statistically significant adjusted MD between groups for women taking herbal medicine plus lifestyle compared with controls in body weight (MD −2.95 kg, 95% CI −4.4 to −1.5, *p* < 0.01) and BMI kg/m^2^ (MD −1.0, 95% CI −1.6 to −0.5, *p* < 0.01) with an estimated large effect size ( $\eta_{p}^{2}$ = 0.12 and 0.11, respectively). Waist circumference was also significantly lower in herbal medicine group (MD −3.4 cm, 95% CI −4.9 to −1.8, *p* < 0.01) with an estimated medium effect size $\left(\eta_{p}^{2}=0.04\right)$. There was no statistically significant difference between groups for waist‐to‐hip ratio (MD 0.001, 95% CI −0.01 to 0.01, *p* = 0.9) (Table 2).

Secondary outcomes relating to reproductive hormonal concentrations for 64 women at 3 months were statistically different for two hormones. Follicular phase oestradiol was increased in the test group (MD 68.9 pmol/L, 95% CI 5.5 to 132.3, *p* = 0.03), and LH was lowered (MD −1.82 IU/L, 95% CI −3.5 to −0.1, *p* = 0.04). An estimated medium effect size was found for both oestradiol and LH ( $\eta_{p}^{2}$ = 0.08 and 0.06, respectively). No significant differences were found between groups for other reproductive hormones including testosterone, SHBG or FAI (Table 2).

Fasting insulin was significantly lower for women taking herbal medicine in addition to lifestyle compared with controls (MD −5.9 mU/L, 95% CI −10.9 to −1.0, *p* = 0.02), estimated as a large effect size $\left(\eta_{p}^{2}=0.1\right)$. No evidence was found for other metabolic outcomes differing between groups (Table 2). Women's use of hypoglycaemic medication (metformin) was reduced at end point with no significant difference between groups (*p* = 0.22). There was a significant improvement for systolic and diastolic blood pressure for women taking the herbal medicine compared with controls (Table 2).

Women assigned to the herbal medicine plus lifestyle group reported significantly improved HRQoL compared with controls for the total PCOSQ score (MD −31.1, 95% CI −41.4 to −20.7, *p* < 0.01) and for the domains of concerns about body hair (MD −3.0, 95% CI −4.8 to −1.1, *p* = 0.01), infertility (MD −3.9, 95% CI −5.7 to −2.1, *p* < 0.01), body weight (MD −5.24, 95% CI −7.8 to −2.7, *p* < 0.01), menstrual problems (MD −3.9, 95% CI −5.3 to −2.4, *p* < 0.01) and emotions (MD −8.4, 95% CI −11.4 to −5.4, *p* < 0.01). The magnitude of effect size was medium to large for concerns about body hair, infertility and body weight and large for the total PCOSQ score, and concerns about the menstrual cycle and emotions (Table 3). Participants taking the herbal medicine tablets plus lifestyle recorded a significantly greater reduction in depression, anxiety and stress scores compared with controls. Mean scores in the test group at end point were −4.3 (95% CI −5.9 to −2.7, *p* < 0.01), −4.0 (95% CI −5.4 to −2.6, *p* < 0.01) and −5.0 (95% CI −6.5 to −3.5, *p* < 0.01), respectively, lower with an estimated large magnitude of effect for all three domains (Table 3).

**Table 3:** Estimated effect of treatment on quality of life (PCOSQ) and psychological morbidity (DASS21)

	Test group (*n* = 60)	Control group (*n* = 62)	Difference between groups at 3 months^a^				
Quality of life: PCOSQ (score range)	Mean	Mean	Adjusted + Mean difference	95% CI		*P* value	Partial eta squared
PCOSQ (score: 25–182)	81.5	109.3	−31.1 (5.2)	−41.4	−20.7	**<0.01**	0.23
Body hair (score: 5–35)	14.9	17.3	−2.98 (0.9)	−4.8	−1.1	**0.02**	0.08
Infertility (score:5–28)	14.6	16.6	−3.9 (0.9)	−5.7	−2.1	**<0.01**	0.13
Body weight (score: 5–35)	22.2	25.8	−5.24 (1.3)	−7.8	−2.7	**<0.01**	0.13
Menstrual problems (score: 5–28)	12.8	17.2	−3.9 (0.7)	−5.3	−2.4	**<0.01**	0.20
Emotions (score: 5–56)	24.2	30.6	−8.4 (1.5)	−11.4	−5.4	**<0.01**	0.21
Psychological morbidity: DASS 21							
Depression 0–21	3.5	7.5	−4.3 (0.8)	−5.9	−2.7	**<0.01**	0.20
Anxiety 0–21	2.4	6.3	−4.0 (0.7)	−5.4	−2.6	**<0.01**	0.22
Stress 0–21	4.9	9.6	−5.0 (0.8)	−6.5	−3.5	**<0.01**	0.27

The PCOSQ was scored with lower scores indicative of better quality of life. This was reversed conventional scoring.

a All results are reported after adjustment for variation in baseline values using ANCOVA.

The residual effect of loss of body weight on the primary outcome (number of days in the menstrual cycle) after controlling for baseline menstrual variation was not significant (*p* = 0.07).

Conception rates were significantly higher for women taking herbal medicine plus lifestyle compared with controls (RR 3.9, 95% CI 1.1 to 13.1, *p* = 0.01). Medically assisted reproduction was not used by any participants during the trial. Pregnant women miscarried at similar rates in the test (4 of 11, 36%) and control groups (1 of 3, 33%), although no conclusions are drawn from such a small number of events. The live birth rate for women wanting to conceive (*n* = 70) was not statistically different based on this sample size (*p* = 0.06).

Two women were withdrawn from the trial with adverse events due to the herbal medicine. Physical symptoms were mild and resolved following cessation of taking the herbal tablets. One participant experienced flu‐like symptoms (headache, lethargy and joint pain) that were relieved following cessation of the herbal tablets. Recommencement of tablets was associated with the return of symptoms. The second adverse event involved abnormal uterine bleeding. At week 5, the participant's menstrual period commenced and continued for 21 days. Although serum concentration of oestradiol, FSH, LH, SHBG, glucose and insulin was within normal ranges, the participant's menstrual period continued for 1 week post cessation of the tablets and subsequently returned to baseline regularity. This participant was withdrawn from the trial at week 6. Overall compliance with the herbal tablets was very high; all but two participants returned empty bottles. One woman returned 17 of the herbal combination tablet, and another returned 12 of the Tribulus Forte tablets. Both of these women had taken less than the prescribed dose.

## Discussion

This RCT demonstrated a statistical and clinically significant improvement for a lifestyle intervention following the addition of a novel herbal combination compared with lifestyle intervention alone with reducing PCOS symptomology including a reduction in the number of days of the menstrual cycle (a large treatment effect on oligomenorrhoea/amenorrhoea), improved anthropometry, oestradiol and LH, fasting insulin, blood pressure, conception rates and quality of life. The study also showed important statistically significant reductions to women's depression, anxiety and stress scores. The lifestyle intervention was not improved by the addition of herbal medicine for biochemical hyperandrogenism including testosterone, SHBG or FAI, gonadotropin hormones FSH or the FSH LH ratio, metabolic measurements of fasting glucose or insulin sensitivity or anthropometric hip‐to‐waist ratio.

The findings from this study compare favourably with published data comparing metformin and the oral contraceptive pill Diane Nova (ethinyl oestradiol 35 μg and cyproterone acetate 2 mg) for menstrual regularity over 3 months (Morin‐Papunen *et al*., 2000). In that RCT, 35 obese women (BMI ≥27) with PCOS taking metformin demonstrated a mean reduction in the number of days in the menstrual cycle from 98 to 74 days (25%, *p* < 0.05) with significant improvements in waist‐to‐hip ratio and fasting insulin (Morin‐Papunen *et al*., 2000). Diane Nova was shown to be more efficient at menstrual cycle regulation with a mean reduction from 88 to 28 days (68%, *p* < 0.05), but no significant impact on anthropometric or metabolic outcomes (Morin‐Papunen *et al*., 2000). Similar reduced menstrual cyclicity was demonstrated in women taking herbal supplements and lifestyle intervention in our study, with a reduction in mean days between cycles from 106 to 62 days (reduction of 44 days in the test group at 3 months, 39%, *p* < 0.01). Use of combined lifestyle and the herbal supplement showed greater reductions of diastolic and systolic blood pressure compared with the control group in this study. Because the oral contraceptive pill has been shown to increase blood pressure and cardiovascular disease risk in young women, (Costello *et al*., 2007, Hickson *et al*., 2011) avoidance of this medication and concomitant reduction in a cardiovascular risk factor for overweight women with PCOS by using combined lifestyle and herbal medication may confer additional health advantages.

This RCT of lifestyle plus herbal medicine demonstrates significant anthropometric improvements for overweight women with PCOS. According to an Australian report into obesity, most women in this study would be classified as having class 2 obesity, a group at severe risk of obesity‐related health conditions (Australian Government Report 2005). The anthropometric improvements for participants in the test group reclassified for many of these women to class 1, with a moderate risk for developing obesity‐related health complications. In light of the limitations for lifestyle intervention for overweight women with PCOS (Moran *et al*., 2011), such marked changes over the study duration with both specific gynaecological and mental health improvements are an important finding.

There were no serious adverse events during the trial, and non‐serious adverse effects were substantially fewer compared with the pharmaceutical interventions clomiphene and metformin for managing PCOS (Legro *et al*., 2007a, 2007b, Tang *et al*., 2012). From reported data, side effects for clomiphene include abdominal pain in 53% of women, diarrhoea 23%, nausea 39%, hot flushes 28%, headaches 44% and mood swings 15%. For metformin, reported side effects include abdominal pain 59%, diarrhoea 65%, nausea 62%, vomiting 30%, headaches 42% and mood swings 17%. Data from these studies report four serious adverse events including one death. Our study did not directly assess these pharmaceutical agents against the herbal medicine combination; however, the side effect profile appears satisfactory, and future studies comparing the effectiveness of the herbal formulation against pharmaceuticals are warranted given the significant improvement in menstrual and reproductive outcomes.

Menstrual cycle regulation from a lifestyle intervention in overweight women with PCOS is consistently associated with loss of body weight (Thomson *et al*., 2008, Moran *et al*., 2013a, 2013b). We found that after correcting for changes in baseline cycle length, changes in bodyweight could only explain 3% of residual variation in menstrual cycle length (*p* = 0.07); however, being assigned to the herbal medicine plus lifestyle group explained 11% of residual variation (*p* < 0.01). This suggests that the impact of the combination on oligomenorrhoea was more complex than being explained by loss of body weight alone.

### Limitations

The lack of a placebo group prevents identification of the active component of this herbal and lifestyle intervention that has generated these outcomes. There was also limited opportunity to control for non‐specific effects that occurred within the trial that may not be attributable to the herbal medicine or the lifestyle intervention including the placebo effect of the herbal tablets and the unintended motivational role of the two consultations provided to women in the combination group to assess well‐being and monitor adverse effects. This RCT utilized a pragmatic research design to answer the research question about clinical effectiveness (Rothwell, 2005, Fønnebø *et al*., 2007, Zwarenstein *et al*., 2008). Research is now needed to examine the discrete effects of components of the interventions and to examine the relationships between herbal medicine and lifestyle intervention.

The lack of blinding could have influenced the estimated treatment effect size particularly for subjective outcomes such as self‐reported QoL, psychology and compliance although objectively measured outcomes were less likely to be at high risk of performance bias (Teixeira *et al*., 2005, Wood *et al*., 2008). The positive impact of reduced body weight on reproductive, metabolic and psychological outcomes in overweight women with PCOS was a likely contributor to the positive outcomes, and further research differentiating the effects of the herbal medicine and reduced body weight may further explain the mechanism of the herbal medicine separately to the effects of lifestyle intervention. This would be best achieved using efficacy RCT design (double‐blinded placebo controlled) to explain the factors of the intervention that may have influenced the outcomes and to validate the size of the treatment effects.

The effectiveness of combined lifestyle and herbal medicine for menstrual regulation and secondary outcomes compared with lifestyle alone was demonstrated over three months, further investigations over longer durations are needed to examine the sustainability of the outcomes.

## Conclusion

This novel trial provides evidence of effectiveness and safety of the combined herbal medicine and lifestyle intervention in overweight women with PCOS. Future research is needed to explain the specific clinical effects of each component of the intervention.

## Authors Contributions

Susan Arentz is a PhD research student at the National Institute of Complementary Medicine, Western Sydney University, Australia. Professor Caroline Smith, A/Professor Jason Abbott and Professor Alan Bensoussan formed her supervisory panel and provided supervisory support and critical assessment of the entire research project. Mr Paul Fahey contributed to the concept, design and the critical revision of the statistical methods and analyses. Dr Birinder (Bobby) Cheema contributed to the concept and design of the lifestyle intervention and intellectually through translation of the evidence‐based guidelines into practice. All authors were integral in the conception and design of the project and contributed critical revision. All authors read the final version of the article and approved.

## Details of Ethical Approval

The study was approved by the Western Sydney University Human Research Ethics Committee (EC00314) on 20 December 2011. An amendment to the trial protocol was approved on 9 August 2012. The reference number was H9407.

## Data Sharing Statement

Participants gave informed consent for anonymized data sharing. The full data set is available on reasonable request from the corresponding author at s.arentz@westernsydney.edu.au.

## Supporting information

Data S1. Supporting Information

Data S2. Supplementary File
Supplementary Table 1. PCOS trial tablet
Supplementary Table 2. MediHerb Tribulus Forte tablet

## References

[ptr5858-bib-0001] AAPCOS (2011). Evidence‐based guideline for the assessment and management of polycystic ovary syndrome. NHMRC. Melbourne, Australia, Jean Hailes Foundation for Women's Health on behalf of the PCOS Australian Alliance.

[ptr5858-bib-0002] Therapeutic Goods Administration . 2000 Notice for Guidance on Clinical Safety Data Management: Definitions and Standards for Expidited Reporting (CPMP/ICH/377/95) Department of Health and Aging. Canberra: Australia, Commonwealth Government.

[ptr5858-bib-0003] Arentz S , Smith CA , Abbott JA , *et al.* 2014a Herbal medicine for the management of polycystic ovary syndrome (PCOS) and associated oligo/amenorrhoea and hyperandrogenism; a review of the laboratory evidence for effects with corroborative clinical findings. BMC Complementary and Alternative Medicine 14(1): 511. 25524718 10.1186/1472-6882-14-511 PMC4528347

[ptr5858-bib-0004] Arentz S , Smith CA , Abbott JA , *et al.* 2014b A survey of the use of complementary medicine by a self‐selected community group of Australian women with polycystic ovary syndrome. BMC Complementary and Alternative Medicine 14(1): 472. 25481654 10.1186/1472-6882-14-472 PMC4265410

[ptr5858-bib-0005] Armanini D , Mattarello MJ , Fiore C , *et al.* 2004 Licorice reduces serum testosterone in healthy women. Steroids 69(11–12): 763–766. 15579328 10.1016/j.steroids.2004.09.005

[ptr5858-bib-0006] Armanini D , Castello R , Scaroni C , *et al.* 2007 Treatment of polycystic ovary syndrome with spironolactone plus licorice. European Journal of Obstetrics and Gynecology and Reproductive Biology 131(1): 61–67. 17113210 10.1016/j.ejogrb.2006.10.013

[ptr5858-bib-0007] Asl MN , Hosseinzadeh H . 2008 Review of pharmacological effects of glycyrrhiza sp. and its bioactive compounds. Phytotherapy Research 22(6): 709–724. 18446848 10.1002/ptr.2362 PMC7167813

[ptr5858-bib-0008] Australian government report (2005). A healthy and active Australia. Health Department. Canberra, Australia. 2014: Government document.

[ptr5858-bib-0009] Azziz R , Marin C , Hoq L , Badamgarav E , Song P . 2005 Health care‐related economic burden of the polycystic ovary syndrome during the reproductive life span. The Journal of Clinical Endocrinology and Metabolism 90(8): 4650–4658. 15944216 10.1210/jc.2005-0628

[ptr5858-bib-0010] Bailey C , Day C . 2004 Metformin: its botanical background. Practical Diabetes International 21(3): 115–117.

[ptr5858-bib-0011] Bone K , Morgan M . 1996 Clinical Applications of Ayurvedic and Chinese Herbs: Monographs for the Western Herbal Practitioner. Phytotherapy Press: Queensland, Australia.

[ptr5858-bib-0012] Chan CC , Koo MW , Ng EH , Tang OS , Yeung WS , Ho PC . 2006 Effects of Chinese green tea on weight, and hormonal and biochemical profiles in obese patients with polycystic ovary syndrome‐‐a randomized placebo‐controlled trial. Journal of the Society for Gynecologic Investigation 13(1): 63–68. 16378915 10.1016/j.jsgi.2005.10.006

[ptr5858-bib-0013] Cohen J . 1973 Eta‐squared and partial eta‐squared in fixed factor ANOVA designs. Educational and Psychological Measurement .

[ptr5858-bib-0014] Costello M , Shrestha B , Eden J , Johnson N , Moran LJ . 2007 Insulin‐sensitising Drugs versus the Combined Oral Contraceptive Pill for Hirsutism, Acne and Risk of Diabetes, Cardiovascular Disease, and Endometrial Cancer in Polycystic Ovary Syndrome. The Cochrane Library. 10.1002/14651858.CD005552.pub2 17253562

[ptr5858-bib-0015] Cronin L , Guyatt G , Griffith L , Wong E , Azziz R , Futterweit W , Cook D , Dunaif A . 1998 Development of a Health‐related Quality‐of‐Life Questionnaire (PCOSQ) for Women with Polycystic Ovary Syndrome (PCOS). Journal of Clinical Endocrinology and Metabolism 83(6): 1976–1987. 9626128 10.1210/jcem.83.6.4990

[ptr5858-bib-0016] Diamanti‐Kandarakis E , Baillargeon J , Iuorno MJ , Jakubowicz DJ , Nestler JE . 2003 A modern medical quandary: polycystic ovary syndrome, insulin resistance, and oral contraceptive pills. The Journal of Clinical Endocrinology and Metabolism 88(5): 1927–1932. 12727935 10.1210/jc.2002-021528

[ptr5858-bib-0017] ESHRE . 2004 Revised 2003 consensus on diagnostic criteria and long‐term health risks associated with polycystic ovary syndrome. Fertility and Sterility 81(1): 19–25. 10.1016/j.fertnstert.2003.10.004 14711538

[ptr5858-bib-0018] ESHRE . 2012 Consensus on women's health aspects of polycystic ovary syndrome (PCOS). Human Reproduction 27(1): 14–24. 22147920 10.1093/humrep/der396

[ptr5858-bib-0019] FertilityFriend . (2014). “Fertility friend.” Retrieved 8 July 2014, 2014, from https://www.fertilityfriend.com/.

[ptr5858-bib-0020] Fisher L , Dixon D , Herson J , Frandowski R , Hearron M , Peace K . 1990 Intention to treat in clinical trials. Statistical Issues in Pharmaceutical Drug Development. 331–350.

[ptr5858-bib-0021] Fønnebø V , Grimsgaard S , Walach H , *et al.* 2007 Researching complementary and alternative treatments–the gatekeepers are not at home. BMC Medical Research Methodology 7(1): 7. 17291355 10.1186/1471-2288-7-7 PMC1800863

[ptr5858-bib-0022] Gaster B , Holroyd J . 2000 St John's Wort for Depression: a systematic review. Archives of Internal Medicine 160(2): 152. 10647752 10.1001/archinte.160.2.152

[ptr5858-bib-0023] Grant P . 2010 Spearmint herbal tea has significant anti‐androgen effects in polycystic ovarian syndrome. A randomized controlled trial. Phytotherapy Research 24(2): 186–188. 19585478 10.1002/ptr.2900

[ptr5858-bib-0024] Greenlee H , Atkinson C , Stanczyk FZ , Lampe JW . 2007 A pilot and feasibility study on the effects of naturopathic botanical and dietary interventions on sex steroid hormone metabolism in premenopausal women. Cancer Epidemiology Biomarkers and Prevention 16(8): 1601–1609. 10.1158/1055-9965.EPI-06-0938 17684134

[ptr5858-bib-0025] Hickson SS , Miles KL , McDonnell BJ , Cockcroft JR , Wilkinson IB , McEniery CM . 2011 Use of the oral contraceptive pill is associated with increased large artery stiffness in young women: the ENIGMA study. Journal of Hypertension 29(6): 1155–1159. 21505350 10.1097/HJH.0b013e328346a5af

[ptr5858-bib-0026] Hoeger KM , Kochman L , Wixom N , Craig K , Miller RK , Guzick DS . 2004 A randomized, 48‐week, placebo‐controlled trial of intensive lifestyle modification and/or metformin therapy in overweight women with polycystic ovary syndrome: a pilot study. Fertility and Sterility 82(2): 421–429. 15302293 10.1016/j.fertnstert.2004.02.104

[ptr5858-bib-0027] IBM (2013). SPSS version 21**:** statistics software

[ptr5858-bib-0028] Izzo AA , Hoon‐Kim S , Radhakrishnan R , Williamson EM . 2016 A critical approach to evaluating clinical efficacy, adverse events and drug interactions of herbal remedies. Phytotherapy Research 30(5): 691–700. 26887532 10.1002/ptr.5591

[ptr5858-bib-0029] Kamel HH . 2013 Role of phyto‐oestrogens in ovulation induction in women with polycystic ovarian syndrome. European Journal of Obstetrics and Gynecology and Reproductive Biology 168(1): 60–63. 23347605 10.1016/j.ejogrb.2012.12.025

[ptr5858-bib-0030] Katz A , Nambi SS , Mather K , *et al.* 2000 Quantitative Insulin Sensitivity Check Index: a simple, accurate method for assessing insulin sensitivity in humans. The Journal of Clinical Endocrinology and Metabolism 85(7): 2402–2410. 10902785 10.1210/jcem.85.7.6661

[ptr5858-bib-0031] Kort DH , Lobo RA . 2014 Preliminary evidence that cinnamon improves menstrual cyclicity in women with polycystic ovary syndrome: a randomized controlled trial. American Journal of Obstetrics and Gynecology 211(5): 487. e481–487. e486. 24813595 10.1016/j.ajog.2014.05.009

[ptr5858-bib-0032] Lai L , Flower A , Prescott P , Wing T , Moore M , Lewith G . 2017 Standardised versus individualised multiherb Chinese herbal medicine for oligomenorrhoea and amenorrhoea in polycystic ovary syndrome: a randomised feasibility and pilot study in the UK. BMJ Open 7(2): e011709 https://doi.org/10.1136/bmjopen-2016-011709 10.1136/bmjopen-2016-011709 PMC5293993 28159846

[ptr5858-bib-0033] Legro RS , Barnhart HX , Schlaff WD , *et al.* 2007a Clomiphene, metformin, or both for infertility in the polycystic ovary syndrome. New England Journal of Medicine 356(6): 551–566. 17287476 10.1056/NEJMoa063971

[ptr5858-bib-0034] Legro RS , Zaino RJ , Demers LM , *et al.* 2007b The effects of metformin and rosiglitazone, alone and in combination, on the ovary and endometrium in polycystic ovary syndrome. American Journal of Obstetrics and Gynecology 196(4): 402.e401–402.e411. 17403436 10.1016/j.ajog.2006.12.025

[ptr5858-bib-0035] Linde, K. , M. M. Berner and L. Kriston (2008). St John's Wort for Major Depression. The Cochrane Library. 10.1002/14651858.CD000448.pub3 PMC7032678 18843608

[ptr5858-bib-0036] Lovibond, S. H. , Lovibond, P.F. (1995). Manual for the Depressive, Anxiety, Stress Scales. Sydney, Psychological Foundation.

[ptr5858-bib-0037] March WA , Moore VM , Willson KJ , Phillips DIW , Norman RJ , Davies MJ . 2010 The prevalence of polycystic ovary syndrome in a community sample assessed under contrasting diagnostic criteria. Human Reproduction 25(2): 544–551. 19910321 10.1093/humrep/dep399

[ptr5858-bib-0038] Marsh KA , Steinbeck KS , Atkinson FS , Petocz P , Brand‐Miller JC . 2010 Effect of a low glycemic index compared with a conventional healthy diet on polycystic ovary syndrome. The American Journal of Clinical Nutrition 92(1): 83–92. 20484445 10.3945/ajcn.2010.29261

[ptr5858-bib-0039] McDonald SM , Liu J , Wilcox S , Lau EY , Archer E . 2016 Does dose matter in reducing gestational weight gain in exercise interventions? A systematic review of literature. Journal of Science and Medicine in Sport 19(4): 323–335. 25846125 10.1016/j.jsams.2015.03.004 PMC4583795

[ptr5858-bib-0040] Mills S , Bone K . 2000 Principles and Practices of Phytotherapy Modern Herbal Medicine. Harcourt Publishers: London.

[ptr5858-bib-0041] Moran LJ , Hutchison SK , Norman RJ , Teede HJ . 2011 Lifestyle changes in women with polycystic ovary syndrome. Cochrane Database Syst Rev 2: CD007506. 10.1002/14651858.CD007506.pub4 PMC6438659 30921477

[ptr5858-bib-0042] Moran LJ , Ko H , Misso M , *et al.* 2013a Dietary composition in the treatment of polycystic ovary syndrome: a systematic review to inform evidence‐based guidelines. Journal of the Academy of Nutrition and Dietetics 113(4): 520–545. 23420000 10.1016/j.jand.2012.11.018

[ptr5858-bib-0043] Moran L , Ranasinha S , Zoungas S , McNaughton S , Brown W , Teede H . 2013b The contribution of diet, physical activity and sedentary behaviour to body mass index in women with and without polycystic ovary syndrome. Human Reproduction https://doi.org/10.1093/humrep/det256 10.1093/humrep/det256 23771201

[ptr5858-bib-0044] Morin‐Papunen LC , Vauhkonen I , Koivunen RM , Ruokonen A , Martikainen HK , Tapanainen JS . 2000 Endocrine and metabolic effects of metformin versus ethinyl estradiol‐cyproterone acetate in obese women with polycystic ovary syndrome: a randomized study 1. The Journal of Clinical Endocrinology and Metabolism 85(9): 3161–3168. 10999803 10.1210/jcem.85.9.6792

[ptr5858-bib-0045] NPAAC (2013). Guidelines for approved pathology collection centres (requirements for medical pathology specimen collection) D. o. H. National Pathology Acreditation Advisory Council, Australian Federal Government.

[ptr5858-bib-0046] Qublan HS , Yannakoula EK , Al‐Qudah MA , El‐Uri FI . 2007 Dietary intervention versus metformin to improve the reproductive outcome in women with polycystic ovary syndrome. A prospective comparative study. Saudi Medical Journal 1694–1699. 17965792

[ptr5858-bib-0047] Rothwell PM . 2005 External validity of randomised controlled trials:“to whom do the results of this trial apply?”. The Lancet 365(9453): 82–93. 10.1016/S0140-6736(04)17670-8 15639683

[ptr5858-bib-0048] Sarris J , Wardle J . 2010 Clinical Naturopathy: An Evidence‐based Guide to Practice. Elsevier: Australia.

[ptr5858-bib-0049] Shahin AY , Mohammed SA . 2014 Adding the phytoestrogen *Cimicifugae Racemosae* to clomiphene induction cycles with timed intercourse in polycystic ovary syndrome improves cycle outcomes and pregnancy rates – a randomized trial. Gynecological Endocrinology 30(7): 505–510. 24592984 10.3109/09513590.2014.895983

[ptr5858-bib-0050] Sills ES , Perloe M , Tucker MJ , Kaplan CR , Genton MG , Schattman GL . 2001 Diagnostic and treatment characteristics of polycystic ovary syndrome: descriptive measurements of patient perception and awareness from 657 confidential self‐reports. BMC Women's Health 1(1): 3. 11545683 10.1186/1472-6874-1-3 PMC55341

[ptr5858-bib-0051] Singh, S. , Kooy, C. , Der Kroy, F . (2013). Chemical characterisation of a herbal formulation for the treatment of polycystic ovary syndrome. Academic thesis (Bachelor of Medical Science Honours 1A), University of Western Sydney.

[ptr5858-bib-0052] Takahashi K , Kitao M . 1994 Effect of TJ‐68 (shakuyaku‐kanzo‐to) on polycystic ovarian disease. International Journal of Fertility and Menopausal Studies 39(2): 69. 8012442

[ptr5858-bib-0053] Tang T , Lord JM , Norman RJ , Yasmin E , Balen AH . 2012 Insulin‐sensitising drugs (metformin, rosiglitazone, pioglitazone, D‐chiro‐inositol) for women with polycystic ovary syndrome, oligo amenorrhoea and subfertility. Cochrane Database Syst Rev 5 Issue (5). Art. No.: CD003053. https://doi.org/10.1002/14651858 10.1002/14651858.CD003053.pub5 22592687

[ptr5858-bib-0054] Teede H , Deeks A , Moran L . 2010 Polycystic ovary syndrome: a complex condition with psychological, reproductive and metabolic manifestations that impacts on health across the lifespan. BMC Medicine 8(1): 41. 20591140 10.1186/1741-7015-8-41 PMC2909929

[ptr5858-bib-0055] Teixeira P , Going S , Sardinha L , Lohman T . 2005 A review of psychosocial pre‐treatment predictors of weight control. Obesity Reviews 6(1): 43–65. 15655038 10.1111/j.1467-789X.2005.00166.x

[ptr5858-bib-0056] Thomson RL , Buckley JD , Noakes M , Clifton PM , Norman RJ , Brinkworth GD . 2008 The effect of a hypocaloric diet with and without exercise training on body composition, cardiometabolic risk profile, and reproductive function in overweight and obese women with polycystic ovary syndrome. Journal of Clinical Endocrinology and Metabolism 93(9): 3373–3380. 18583464 10.1210/jc.2008-0751

[ptr5858-bib-0057] Vickers A , Zollman C . 1999 Herbal medicine. BMJ 319(7216): 1050–1053. 10521203 10.1136/bmj.319.7216.1050 PMC1116847

[ptr5858-bib-0058] Wang JG , Anderson RA , Graham GM 3rd , *et al.* 2007 The effect of cinnamon extract on insulin resistance parameters in polycystic ovary syndrome: a pilot study. Fertility and Sterility 88(1): 240–243. 17296187 10.1016/j.fertnstert.2006.11.082

[ptr5858-bib-0059] Wardle J , Lui CW , Adams J . 2012 Complementary and alternative medicine in rural communities: current research and future directions. The Journal of Rural Health 28(1): 101–112. 22236320 10.1111/j.1748-0361.2010.00348.x

[ptr5858-bib-0060] Wood L , Egger M , Gluud LL , *et al.* 2008 Empirical evidence of bias in treatment effect estimates in controlled trials with different interventions and outcomes: meta‐epidemiological study. BMJ 336(7644): 601–605. 18316340 10.1136/bmj.39465.451748.AD PMC2267990

[ptr5858-bib-0061] Yaginuma TI , Izumi R , Yasui H , Arai T , Kawabata M . 1982 Effect of traditional herbal medicine on serum testosterone levels and its induction of regular ovulation in hyperandrogenic and oligomenorrheic women. Nippon Sanka Fujinka Gakkai Zasshi 34(7): 939. 7108310

[ptr5858-bib-0062] Zhang J , Li T , Zhou L , Tang L , Xu L , Wu T , Lim DCE . 2010a Chinese herbal medicine for subfertile women with polycystic ovarian syndrome. The Cochrane Database Of Systematic Reviews 9: CD007535. 10.1002/14651858.CD007535.pub2 20824862

[ptr5858-bib-0063] Zhang J , Li T , Zhou L , Tang L , Xu L , Wu T , Lim DCE . 2010b Chinese herbal medicine for subfertile women with polycystic ovarian syndrome. Cochrane Database of Systematic Reviews DOI https://doi.org/10.1002/14651858.CD007535.pub2. 10.1002/14651858.CD007535.pub2 20824862

[ptr5858-bib-0064] Zwarenstein M , Treweek S , Gagnier JJ , Altman D , Tunis S , Haynes B , Oxman A , Moher D . 2008 Improving the reporting of pragmatic trials: an extension of the CONSORT statement. BMJ 337. 10.1136/bmj.a2390 PMC3266844 19001484

