# Supplementary material for: Combined Lifestyle and Herbal Medicine in Overweight Women with Polycystic Ovary Syndrome (PCOS): A Randomized Controlled Trial
Source: Phytother Res. 2017 Jul 7;31(9):1330–40. doi: 10.1002/ptr.5858 (PMC5599989; doi:10.1002/ptr.5858)
Supplement: Supplementary file 2 — Data S2. Supplementary File Supplementary Table 1. PCOS trial tablet Supplementary Table 2. MediHerb Tribulus Forte tablet [file PTR-31-1330-s002.docx]

**Supplementary File**

This trial was prospectively registered with the Australian and New Zealand Clinical Trials Registry (ANZCTR) on the 25^th^ of January 2012. It can be accessed at <https://www.anzctr.org.au/Trial/Registration/TrialReview.aspx?id=347666&isReview=true>

**Supplementary Tables**

Supplementary Table 1. PCOS trial tablet

| **Species (plant part)** | **Extract equivalent to dry herb (mg) per tablet** | **Extract equivalent to dry herb (mg) per daily dose** |
| --- | --- | --- |
| *Glycyrrhiza glabra* (root) | 750 | 2250 |
| *Paeonia lactiflora* (root) | 750 | 2250 |
| *Cinnamomum verum*  (stem bark) | 750 | 2250 |
| *Hypericum perforatum* (flowering herb) | 750 | 2250 |

Supplementary Table 2. MediHerb Tribulus Forte tablet

| **Species (plant part)** | **Extract equivalent to dry herb (mg) per tablet** | **Extract equivalent to dry herb (mg) per daily dose** |
| --- | --- | --- |
| *Tribulus terrestris* (aerial parts) standardised to furostanol saponins 110 mg per tablet calculated as protodioscin | 13,500 | 40,500 |
| Australian Register of Therapeutic Goods (ARTG): Listed medicines number: 185079  Sponsor: Integria Healthcare Australia Pty Ltd | | |
